# Supplementary material for: Balancing the Strength–Impact Relationship and Other Key Properties in Polypropylene Copolymer–Natural CaSO4 (Anhydrite)-Filled Composites
Source: Int J Mol Sci. 2023 Aug 10;24(16):12659. doi: 10.3390/ijms241612659 (PMC10454880; doi:10.3390/ijms241612659)
Supplement: Supplementary file 1 [file ijms-24-12659-s001.zip › ijms-2454351-supplementary.pdf]

Supplementary Material S1 (online publication)

Support information regarding the manuscript sent for publication to IJMS/MDPI:

Balancing the strength-impact relationship and other key properties in polypropylene copolymer-natural $CaSO_4$ (anhydrite) filled composites.

Marius Murariu [1,2*], **Fouad Laoutid** [1], **Yoann Paint** [1], **Oltea Murariu** [1], **Jean-Marie Raquez** [2] and **Philippe Dubois** [1,2*]

1 Laboratory of Polymeric and Composite Materials, Materia Nova Materials R&D Center & UMONS Innovation Center, 3 Avenue Copernic, 7000-Mons, Belgium; fouad.laoutid@materianova.be (F.L.); yoann.paint@materianova.be (Y.P.); oltea.murariu@materianova.be (O.M.).

2 Laboratory of Polymeric and Composite Materials, Center of Innovation and Research in Materials and Polymers (CIRMAP), University of Mons (UMONS), Place du Parc 20, 7000 Mons, Belgium; jean-marie.raquez@umons.ac.be (J-M.R.).

3 *Correspondence: marius.murariu@materianova.be (M.M.) and philippe.dubois@umons.ac.be (P.D.); Tel.: +32-65-373000 (P.D.).

1. FTIR analysis of ionomeric modified PPc composites produced with internal mixers.

For the evidence of the ionomeric modifications in composites obtained using different methods of production (i.e., with internal mixers for reactive mixing, or using twin-screw extruders (TSE) for reactive extrusion (REx)), representative samples have been characterized by FTIR, as it is discussed in the manuscript. Figure 1 shows the comparative FTIR spectra of the main raw materials (PPc, ZA, and AII) and those of PPc-AII composites (with/without ZA modifier) produced using internal mixers.

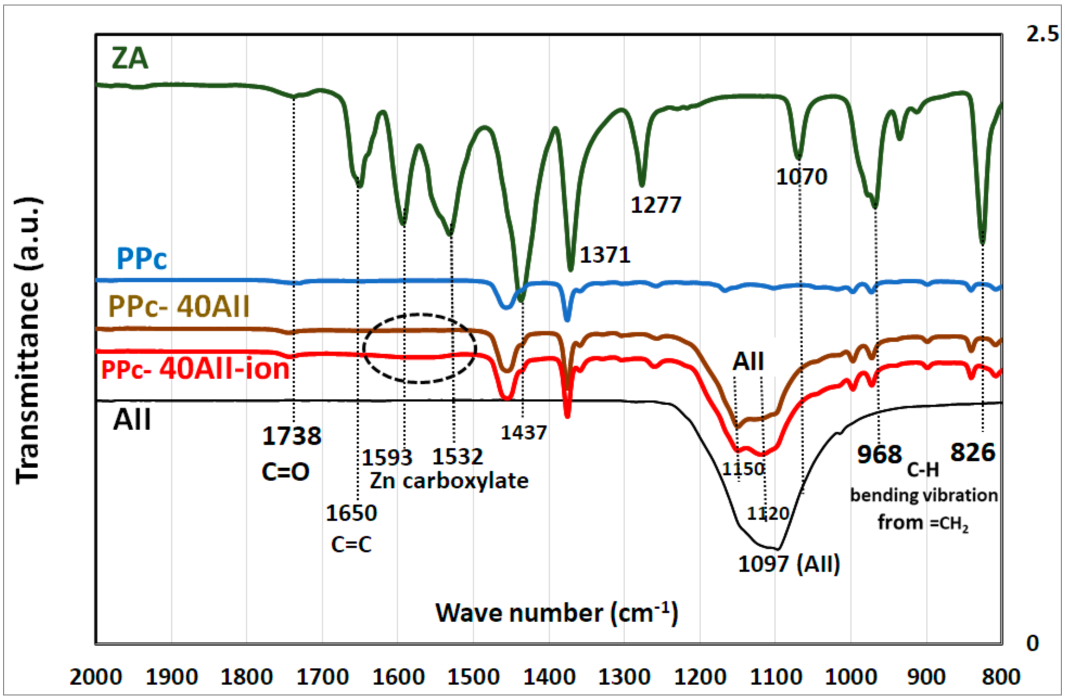

Figure S1. Comparative FTIR spectra of raw materials (PPc, ZA, AII), PPc-40AII composites and of PPc-AII-ion composites obtained by reactive melt-blending using internal mixers.

Abbreviations: PPc: polypropylene copolymer; ZA: zinc diacrylate; AII: $CaSO_4$ (anhydrite II); PPc-40AII: PPc- 40% AII; PPc-40AII-ion: PPc- 40% AII- 2% ZA.

Short comments: It is worth pointing out that the FTIR analysis of composites obtained either with internal mixers or REx led to very similar conclusions. For shortness, only the composites obtained by REx have been discussed in the manuscript, therefore is highly recommended to follow the key aspects and the references already mentioned in the paper. Similarly, for the same wave range (i.e., 1700-800 cm^{-1}) hereinafter we will concern only some general statements connected to the presence of AII and reactive ionomeric modification of PPc/AII with ZA using internal mixers. Still, it's noteworthy mentioning that the reactive modification of PPc with ZA can produce thermoreversible ionic crosslinking, as well as covalent bonds with the polyolefin chains (Scheme 1, from the paper).

Regarding the unfilled PPc, with only one exception (an absorption band around 1260 cm^{-1} ascribed to the presence of an additive, e.g., an antistatic agent), all FTIR signals/bands specific to PP have identified as reported in the prior art (see the manuscript). ZA exhibited multiple characteristic absorption bands as assigned in Fig. S1. It shows a strong absorption band at 1650 cm^{-1} assigned to stretching vibrations of C=C bonds (in conjugation with C=O groups), at 1593 and 1532 cm^{-1} attributed to the asymmetric stretching of Zn carboxylate anion, and at 1437 cm^{-1} assigned to the symmetric stretching of strongly coupled carboxylate anion from ZA. At lower wavenumbers, ZA shows additional absorption bands, e.g., at 1371 cm^{-1} (attributed to the $–CH_2–$ wagging, but also often described as $\delta as(COO^{-})$), at 1277 cm^{-1} (peak assignment to α,β-unsaturated carboxylate), at 968 and 826 cm^{-1} (assignments to out-of-plane C–H bending vibration from $=CH_2$). Analyzing the spectra of PPc-40AII-ion samples produced by reactive melting, the absence or the strong attenuation of the signal at 1650 cm^{-1}, thus assigned to C=C bonds from the bifunctional ZA, is reasonably ascribed to the free-radical reaction of ZA (via its carbon-carbon double bond) and PPc chains mostly through its methine groups, leading to the formation of covalent bonds, i.e., of Zn-ionomer. Moreover, the peaks at 968 and 826 cm^{-1} are not detected anymore after the reactive melt-mixing of PPc with AII/ZA. However, by comparing the spectra of PPc-40AII of PPc-40AII-ion samples in the range from 1600 cm^{-1} to 1500 cm^{-1} (as highlighted by an ellipse drawn in Fig. S1), only a broadening region is seen for the ionomeric modified composites, with fair absorption identified at around 1560 cm^{-1}, that likely can correspond to the carbonyl groups/carboxylate stretching of the zinc carboxylate. However, the loading of ZA in composites is low (i.e., 2 wt.%), therefore, the spectra of PPc-40AII-ion and PPc-40AII composites reveal only a few differences. On the other hand, the spectra of AII shows a broad band above 1100 cm^{-1}, assigned to the presence of $(SO_4)^{2-}$ groups. This strong band is also identified in

PPc/AII composites (around 1120 cm^{-1}), which also show supplementary/distinct peaks at around 1150 cm^{-1}, that are specific for the calcined gypsum at high temperature.

(**References**: please consider the bibliography indicated in manuscript as support for the FTIR section)

2. Rheological investigations on samples produced with twin-screw extruders (TSE)

Traditional rheological measurements (MFR/melt flow rate, or melt flow index/MFI) were performed on key samples using melt flow testers. The MFR was determined on samples produced by extrusion (as granules) following the procedure described in ASTM D1238, using a Davenport 10 Melt Flow Indexer (AMETEK Lloyd Instruments Ltd., West Sussex, UK), at a temperature of 230 °C, with a 2.16 kg load. Table S1 shows selected results regarding the melt fluidity (MFR values) of PPc-AIIt and PPc-AII-ion composites, by comparing to the unfilled PPc.

Table S1. Melt flow rate (MFR at 230 °C, 2.16 kg) of samples produced by extrusion (TSE).

**Sample code**	**Filler, wt.%**	**Processing with TSE**	**MFR, g/10 min**
PPc (processed)	**0**	reference	12.9
PPc-20AIIt	20	Non-reactive melt-compounding, 1% EBS in AII filler	14.1
PPc-40AIIt	40		10.9
PPc-20AII-ion	20	Reactive extrusion (REx) in presence of 2% ZA ionomer	8.4
PPc-40AII-ion	40		3.5

Short comments: For clarity, it is important to add that AIIt denotes AII "treated" with 1% EBS, whereas "AII-ion", was used for the filler/formulations with 2% ZA ionomeric additive, as modified by REx (please consider for more information the manuscript).

First, it is seen that the ionomeric modification of PPC with ZA lead to a more significant increase of melt viscosity (decrease of MFR values), well associated with the amounts of filler. The grafting of ZA on PPc chains by REx and the formation of crosslinked structures, as well as the good interactions with the filler (also known to increase the melt viscosity), can explain in great part the MFR results obtained following the analysis of PPc-AII-ion composites. On the other hand, the lubricant effect of EBS is well evidenced, the MFR of PPc-40AIIt composites (i.e., 10.9 g/cm^3) at 40% filler loading being rather comparable with that of the PPc matrix (12.9 g/cm^3). More comprehensive rheological investigations (viscosity, storage, and loss moduli, etc.) using special rheometers can be further considered in the frame of forthcoming studies.
